# Assessing GFR With Proenkephalin

**DOI:** 10.1016/j.ekir.2023.08.006

**Published:** 2023-08-19

**Authors:** Remi Beunders, Leslie J. Donato, Roger van Groenendael, Birte Arlt, Cristiane Carvalho-Wodarz, Janin Schulte, Anton CC. Coolen, John C. Lieske, Jeffrey W. Meeusen, Allan S. Jaffe, Peter Pickkers

**Affiliations:** 1 Department of Intensive Care Medicine, Radboud University Medical Center, Nijmegen, The Netherlands; 2 Radboud Institute for Molecular Life Sciences (RIMLS), Radboud University Medical Center, Nijmegen, The Netherlands; 3 Department of Laboratory Medicine and Pathology, Mayo Clinic, Rochester, Minnesota, USA; 4 Department of Anesthesiology, Pain and Palliative Medicine, Radboud University Medical Center, Nijmegen, The Netherlands; 5 SphingoTec GmbH, Hennigsdorf, Germany; 6 Department of Biophysics, Donders Institute, Radboud University, Nijmegen, The Netherlands; 7 Saddle Point Science Europe BV, Nijmegen, The Netherlands; 8 Division of Nephrology and Hypertension, Mayo Clinic, Rochester, Minnesota, USA; 9 Department of Cardiology, Mayo Clinic, Rochester, USA

**Keywords:** acute kidney injury, creatinine, estimated glomerular filtration rate, gold standard, kidney function, proenkephalin

## Abstract

**Introduction:**

In clinical practice, kidney (dys)function is monitored through creatinine-based estimations of glomerular filtration rate (eGFR: Modification of Diet in Renal Disease [MDRD], Chronic Kidney Disease Epidemiology Collaboration [CKD-EPI]). Creatinine is recognized as a late and insensitive biomarker of glomerular filtration rate (GFR). The novel biomarker proenkephalin (PENK) may overcome these limitations, but no PENK-based equation for eGFR is currently available. Therefore, we developed and validated a PENK-based equation to assess GFR.

**Methods:**

In this international multicenter study in 1354 stable and critically ill patients, GFR was measured (mGFR) through iohexol or iothalamate clearance. A generalized linear model with sigmoidal nonlinear transfer function was used for equation development in the block-randomized development set. Covariates were selected in a data-driven fashion. The novel equation was assessed for bias, precision (mean ± SD), and accuracy (eGFR percentage within ±30% of mGFR, P30) in the validation set and compared with MDRD and CKD-EPI.

**Results:**

Median mGFR was 61 [44–81] ml/min per 1.73 m^2^. In order of importance, PENK, creatinine, and age were included, and sex or race did not improve performance. The PENK-based equation mean ± SD bias of the mGFR was 0.5 ± 15 ml/min per 1.73 m^2^, significantly less compared with MDRD (8 ± 17, *P* < 0.001) and 2009 CKD-EPI (5 ± 17, *P* < 0.001), not reaching statistical significance compared with 2021 CKD-EPI (1.3 ± 16, *P* = 0.06). The P30 accuracy of the PENK-based equation was 83%, significantly higher compared with MDRD (68%, *P* < 0.001) and 2009 CKD-EPI (76%, *P* < 0.001), similar to 2021 CKD-EPI (80%, *P =* 0.13).

**Conclusion:**

Overall, the PENK-based equation to assess eGFR performed better than most creatinine-based equations without using sex or race.

## Graphical abstract

**Figure:**
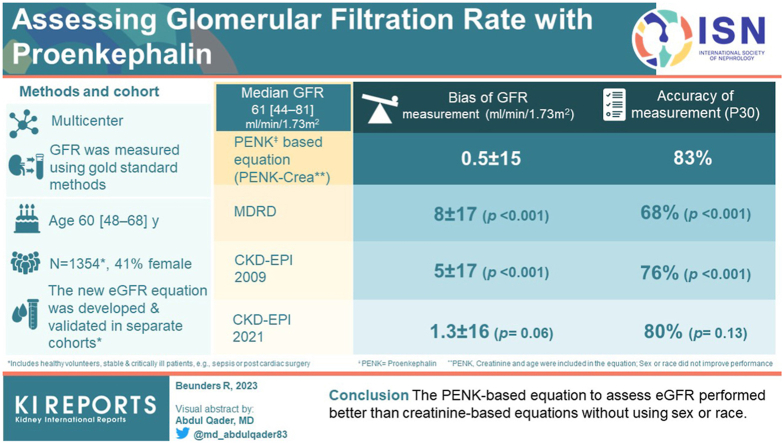

The measurement of creatinine to assess eGFR to represent kidney function in ambulatory and hospitalized patients is a frequently requested diagnostic test. Abnormal kidney function presages adverse clinical outcomes and is an important adjunct to the dosing of renally excreted drugs. Creatinine-based equations, such as MDRD,^1^ 2009 CKD-EPI,^2^ and 2021 CKD-EPI,^3^ are used in daily clinical practice.^4^ However, there are multiple shortcomings in creatinine-based calculations of eGFR, such as muscle mass and hydrational or nutritional status.^5^^,^^6^ In addition, both tubular reabsorption and secretion of creatinine influence the accuracy of creatinine-based eGFR equations.^4^^,^^7^ This may lead to not identifying those at risk of adverse health events.^8^ Finally, the use of race in these equations has been reevaluated,^3^ and implementation of race-free equations was recently recommended.^9^ The true GFR can be measured using the clearance of inulin, iohexol, or iothalamate.^10^ However, these methods require parenteral administration, are labor intensive, and may therefore not be suited for clinical practice. A method to estimate or measure GFR that is more accurate, precise, exempt from bias, and feasible in clinical practice remains an unmet medical need.

PENK, fully: proenkephalin A 119–159 is an endogenous protein (molecular weight ∼4.5 kDa), with no protein binding or cleaving known, that is purely filtered by the glomerulus and not secreted or absorbed in the tubules.^11^ PENK is used as a biomarker of renal function or as a predictor of acute kidney injury.^12^, ^13^, ^14^, ^15^ Many comorbidities that can occur due to or in association with acute kidney injury, such as sepsis, do not seem to influence the association of PENK with GFR.^16^ In a cohort of critically ill patients with septic shock, an excellent correlation was found between plasma PENK with the mGFR using iohexol plasma clearance.^17^ Accordingly, PENK could potentially be used to calculate eGFR which may be more accurate than creatinine-based equations. This study aimed to develop such an equation and assess its performance compared with current creatinine-based equations.

## Methods

### Study Design

Data were collected according to the ethical principles of the Declaration of Helsinki and approved by local medical ethical committees.^17^^,^^18^ Subjects included were healthy and tested before organ donation, or had a stable kidney function which was measured either on an outpatient clinic setting: patients with and without risk factors for chronic kidney disease (CKD) and after organ donation (predominately kidney donation), or were critically ill and admitted to an intensive care unit: post-cardiac surgery and patients diagnosed with having sepsis or septic shock according to the sepsis-3 criteria.^19^ Demographic variable “race” was collected because of the covariate in current creatinine-based equations and was self-reported.

### GFR Assessment

GFR was measured using iohexol plasma clearance or iothalamate renal clearance using the gold standard method of each compound and obtained as part of clinical practice or research purposes.^10^^,^^20^ These compounds were measured using liquid chromatography with tandem mass spectrometry.^21^^,^^22^ Before (baseline) and after i.v. iohexol administration or s.c. iothalamate injection, multiple blood and urine samples were acquired from 0.75 to 6 hours post-administration.^20^^,^^23^^,^^24^ Blood samples were acquired in EDTA-coated tubes after the redistribution phase, during the “slow phase” from 1.5 to 6 hours post-administration, to reconstruct the disappearance curve of iohexol.^20^^,^^23^ The slope-intercept method was used to determine the clearance of the iohexol, and the Bröchner-Mortensen correction was applied to adjust the redistribution phase.^23^^,^^25^ The calculated GFR was then corrected for body surface area.^25^^,^^26^ Iothalamate was injected s.c. after which iothalamate clearance was determined through iothalamate measurement in plasma and urine samples from 0.75 at 1.5 to 2 hours post-administration.^24^ The calculated GFR was then corrected for body surface area.^26^ For comparison between equations, eGFR was calculated using the MDRD,^27^ 2009 CKD-EPI eGFR_Cr_,^28^ and 2021 CKD-EPI eGFR_Cr_^3^ equations.

### Laboratory Measurements

During the GFR assessments, plasma samples were collected for PENK and creatinine concentration measurement using the sphingotest penKid sandwich immunoassay (SphingoTec GmbH, Hennigsdorf, Germany), as described previously.^21^ Blood was drawn in EDTA-coated tubes and was centrifuged before aliquotation of the plasma for storage at −80 °C until batch analysis. Mouse monoclonal anti-PENK 152 to 159 antibodies were coated to white polystyrene microtiter plates as the capture antibody, and mouse monoclonal anti-PENK 129 to 144 antibody labeled with MACN-Acridinium-NHS ester was used as the tracer antibody. The lower detection limit was 7 pmol/l, and the mean interassay coefficient of variation was 5.7% in the measuring range of 10.9 to 686.3 pmol/l. Plasma creatinine concentration was measured in lithium-heparin tubes according to hospital standards in the Mayo Clinic (Rochester, MN, USA) and the Radboud University Medical Center (Nijmegen, The Netherlands) using an IDMS traceable enzymatic assay.

### Equation Development

Apart from plasma PENK and creatinine, additional covariates included sex, age, race (African American, American Indian, Asian American, and White), and patient diagnostic category: potential kidney donor, post-kidney donor, post-kidney transplant recipient, post other than kidney organ recipient, CKD, post-cardiac surgery, and septic (shock) patients. A generalized linear model with a sigmoidal nonlinear transfer function was used for continuous output regression and equation building. The cohort was block randomized into a development and validation set (ratio 1.5:1), ensuring equal distribution of measured eGFR and type of patient over the sets. The validation set was blinded until the equations were determined. SaddlePoint-Signature (version 2.10, London, UK) was used for these analyses. SPSS (version 25, Armonk, NY) and GraphPad Prism (version 5.03, San Diego, CA) were used for statistical tests. Data are presented in medians with interquartile ranges [IQRs], mean ± SD, or frequencies with a 95% CI as appropriate.

### Equation Validation

The newly developed equation was evaluated using a validation data set. The performance was tested in the total validation cohort and in subgroups of stable patients and critically ill patients with sepsis and those with post-cardiac surgery. The equation was assessed for bias, accuracy, and precision, through Bland-Altman analyses. For bias, the mean and predicted-kidney function differences were analyzed, depicted as mGFR minus eGFR (negative bias reflects overestimation), and tested using a paired *t* test. Furthermore, the Lin’s concordance correlation coefficient was used.^29^ For precision, the SD of the differences between true and eGFR was assessed. Bland-Altman analyses were conducted.^30^ For accuracy, the percentage of predicted kidney function between a ±30% range of the true kidney function was determined (P30)^28^ and tested with the McNemar test for paired proportions. To enable clinical decision-making, the 95% and 50% prediction intervals were calculated for which a quantile regression was used to develop a model for the prediction of mGFR at the quantiles 2.5th, 10th, 25th, 50th, 75th, 90th, and 97.5th.^31^ The prediction intervals were calculated as the median difference of the 2.5th and 97.5th quantiles for 95% prediction interval and median difference between the 25th and 75th quantiles for 50% prediction interval. Equation performance was compared with the widely used creatinine-based MDRD^1^ and 2009 CKD-EPI equations.^28^ The race-free 2021 CKD-EPI eGFR_Cr_ equation was published while this analysis was ongoing^3^ and was therefore also compared with the new PENK-based equation. Furthermore, the European Kidney Function Consortium^32^ and revised Lund-Malmö^33^ equations were used for comparison with the PENK equation as secondary endpoints. Statistical significance was accounted to *P* values of <0.05.

## Results

The GFR was measured by gold standard methods (mGFR) in 1354 patients (Table 1). Patients were post-kidney transplant (*n* = 671, stable), post-other organ transplant (*n* = 218, stable), post-cardiac surgery (*n* = 176, in Intensive Care unit), potential kidney donors (*n* = 142, stable), known to have CKD (*n* = 71, stable), post-kidney donation (*n* = 53, stable), and diagnosed with having septic shock (*n* = 23, in Intensive Care unit). Patients had a median [IQR] age of 60 [48–68] years, and 41% were female. Median [IQR] creatinine was 115 [88–142] μmol/l, and median [IQR] mGFR was 61 [44–81] ml/min per 1.73 m^2^. In stable patients, the median [IQR] mGFR was 55 [41–74] ml/min per 1.73 m^2^, whereas in critically ill patients, it was 95 [74–113], illustrating that also patients with a hyperdynamic circulation and increased renal blood flow were included in the critically ill cohorts.^34^ Patients in the validation set had similar baseline demographic characteristics as the development set (Table 1).

**Table 1:** Patient baseline demographic characteristics

Demographics	Total cohort	Development set *n* = 811	Critically ill *n* = 112	Validation set *n* = 543	Critically ill *n* = 87
Subgroup: state of kidney function	*n* = 1354	Stable *n* = 699		Stable *n* = 456	
Age (yrs)	60 [48–68]	59 [47–66]	69 [62–74]	57 [47–66]	65 [59–74]
Gender, male n (%)	794 (59)	373 (53)	95 (85)	251 (55)	75 (86)
Race, n (%)					
African American	25 (2)	18 (3)	1 (1)	9 (2)	
American Indian	11 (1)	6 (1)		5 (1)	
Asian American	30 (2)	16 (2)		14 (3)	
White	1235 (91)	630 (90)	111 (99)	407 (90)	87 (100)
Creatinine (μmol/l)	115 [88–142]	115 [97–150]	83 [70–99]	115 [88–150]	77 [66–93]
Proenkephalin (pmol/l)	76 [55–109]	80 [58–113]	53 [38–68]	85 [61–114]	53 [40–62]
Measured GFR (ml/min/1.73 m^2^)	61 [44–81]	57 [42–75]	94 [69–111]	55 [41–74]	95 [74–113]

GFR, glomerular filtration rate.

Data are presented as frequency (percentage) or median [interquartile range].

GFR was measured using gold standard iothalamate or iohexol clearance.

Conversion factors for units: serum creatinine in mg/dl to μmol/l, ×88.42.

### Equation Development

**Figure 1:**
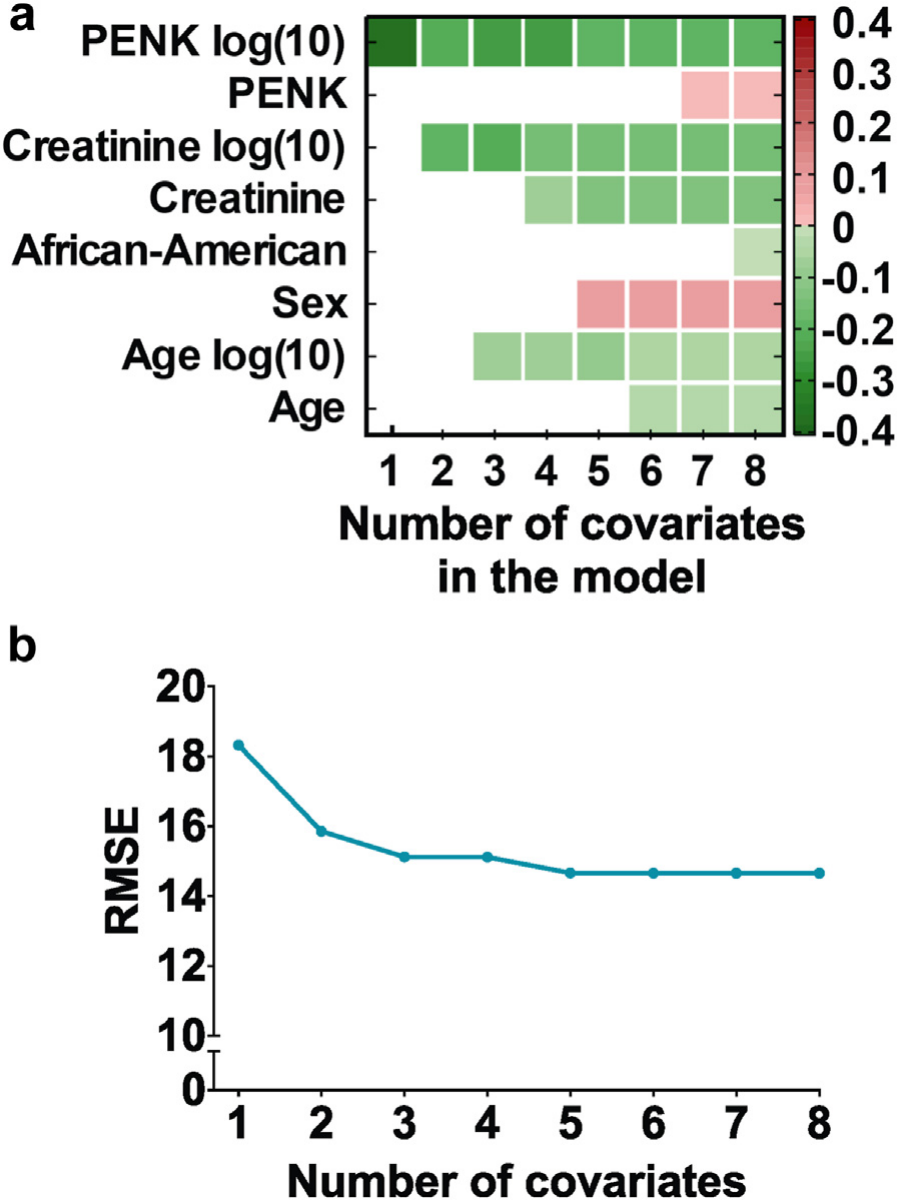
Equation development. (a) Figure with on the y-axis eight different covariates, with their color representing the β-coefficient as depicted in the legend. On the x-axis is the number of the covariate in the model. A higher coefficient represents a higher positive correlation of the covariate with the GFR. In a stepwise iterative removal method, covariates were first all included in the model and then removed in the sequence of importance, starting with the lowest contribution to the model's performance (African-American). The most important covariates were log(10) proenkephalin, log(10) creatinine, and age log(10). (b) RMSE (Root Mean Square Error) of the model with on the x-axis the number of covariates on the model. The covariates were added in a stepwise-enter method, in the sequence of importance, starting with log(10) proenkephalin. A lower RMSE value illustrates a more accurate predicting model. After adding covariate log (10) proenkephalin, log(10) creatinine, and log(10) age, the RMSE does not significantly improve any further. PENK, proenkephalin.

Log(10) PENK had a Pearson’s correlation coefficient of −0.76 (95% CI −79 to −73) (*P* < 0.001) with the mGFR, compared with −0.75 (−78 to −72) (*P* < 0.001) for log(10) creatinine (Supplementary Figure S1). Pearson’s correlation coefficient with the mGFR was 0.79 (*P* < 0.001) for MDRD, 0.84 (*P* < 0.001) for 2009 CKD-EPI, and 0.85 (*P* < 0.001) for 2021 CKD-EPI. Apart from PENK, available variables were tested through bootstrap iterations, using absolute and logarithmic (10) representation of continuous variables. The correlation coefficient of proenkephalin with the mGFR was 0.76, followed by creatinine at 0.75 and age at 0.17 (Table 2). Logarithmic representation of the data resulted in the most accurate equations according to SaddlePoint-Signature output. In a model with all variables, PENK was last removed using a stepwise iterative removal method, indicating that it is the most important variable (Figure 1a). Thereafter, the root mean square error was tested using a stepwise iterative introduction method; PENK narrowed the root mean square error most significantly, followed by creatinine and age (Figure 1b). Again, indicating that PENK is the most important variable for the eGFR equation building. Nominal covariates describing “race” and “age” did not further improve the performance of the equations. Regression models were applied using a linear and nonlinear form with input or output noise. The optimal model used a sigmoidal nonlinear transfer function and considered output noise. The covariates included in the equation in order of importance were as follows: PENK, creatinine, and age, all in log(10) (Figure 1). See Table 2 for the PENK-Crea equation, together with the PENK-only equation for situations when creatinine is not available, measurable, or representative, for example, in hyperbilirubinemia, neuromuscular diseases, muscle atrophy, or amputees.

**Table 2:** Covariates and the PENK-Crea equation

Covariate	Pearson’s correlation coefficient *r*
Proenkephalin log(10)	−0.76
Creatinine log(10)	−0.75
Creatinine	−0.67
Proenkephalin	−0.64
Age log(10)	−0.17
Age	−0.17

PENK-Crea and PENK-only equation.

eGFR_PENK-Crea_ = 72.492926 ∗ tanh (5.489828 − 0.630472∗Age_log10 − 1.286274 ∗ Creatinine_log10 − 1.100716 ∗ Proenkephalin_log10) + 84.176451

eGFR_PENK_ = 10^ˆ^[3.79 − 0.777∗log(PENK) − 0.324∗log(Age)]

For the equations, creatinine was used in the μmol/l format. For use with mg/dl, multiply creatinine values with 88.42 before log(10) transformation. The PENK-only equation can be used when creatinine is not available, measurable or representative, e.g., in hyperbilirubinemia, neuromuscular diseases, muscle atrophy or in amputees, see supplement results table S1 for the performance of the PENK-only equation.

### Equation Validation

A new equation (Proenkephalin-Creatinine eGFR, PENK-Crea) was developed using a combined group of all stable and critically ill patients (*n* = 811). The equation was tested for performance in the total validation set (*n* = 543) and separately in validation subgroups of stable patients (*n* = 456) and critically ill patients (*n* = 87). Contour plots illustrate the impact of each parameter in the PENK-Crea equation on the calculated eGFR, compared with 2021 CKD-EPI (Supplementary Figures S2 and S3). See Supplementary Table S1 for the performance of the PENK-only, the European Kidney Function Consortium^32^ and revised Lund-Malmö^33^ equations.

#### Validation in All Patients

**Figure 2:**
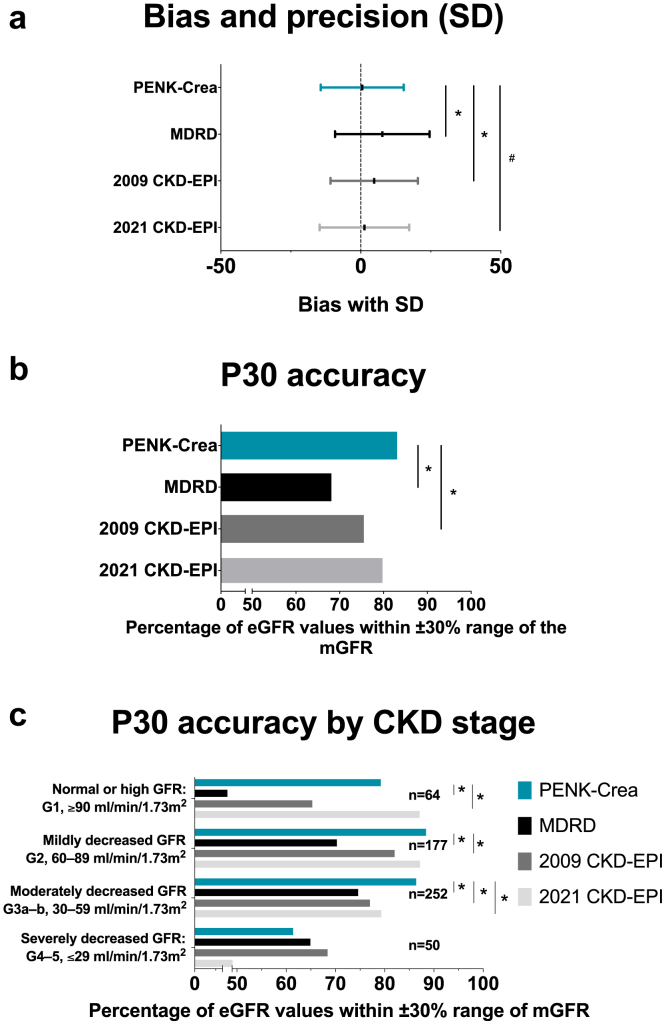
An overview of the performance of the PENK-Crea and the MDRD and CKD-EPI equations. (a) Mean bias in ml/min/1.73 m^2^ with SD from the measured GFR (mGFR) of all equations. The estimations of the GFR calculated with PENK-Crea had a significantly lower mean±SD bias compared to the mGFR than the MDRD: *P* < 0.001 and 2009 CKD-EPI: *P* < 0.001, and a borderline significant difference with the 2021 CKD-EPI (*P* = 0.06). (b) P30 accuracy (proportion of estimated GFR (eGFR) that is within ±30% of the mGFR) of the 3 equations. When using the PENK-Crea, the GFR estimations were significantly more accurate compared to the MDRD (*P* < 0.001) and 2009 CKD-EPI (*P* < 0.001), not to the 2021 CKD-EPI (*P* = 0.13). (c) The P30 accuracy (proportion of eGFR that is within ±30% of the measured GFR) of the PENK-Crea equation compared to eGFR based on MDRD and CKD-EPI equations. The patients are categorized on their mGFR using the KDIGO CKD classification, which was combined to prevent small groups. PENK-Crea had a higher accuracy in the category “G1, ≥90 ml/min/1.73 m^2^” compared to MDRD (*P* < 0.001), 2009 CKD-EPI (*P* = 0.02), and similar to the 2021 CKD-EPI (*P* = 0.10). PENK-Crea had a higher accuracy in the category “G2, 60–89 ml/min/1.73 m^2^” compared to MDRD (*P* < 0.001), 2009 CKD-EPI (*P* = 0.04), and similar to 2021 CKD-EPI (*P* = 0.86). PENK-Crea had the highest accuracy in the category “G3a–b, 30–59 ml/min/1.73 m^2^” (MDRD: *P* < 0.001, 2009 CKD-EPI: *P* = 0.002 and 2021 CKD-EPI: *P* = 0.03). In categories “G4–5, ≤29 ml/min/1.73 m^2^,” there was no statistical difference (MDRD: *P* = 0.77, 2009 CKD-EPI: *P* = 0.39, and 2021 CKD-EPI: *P* = 0.09). GFR, glomerular filtration rate.

**Figure 3:**
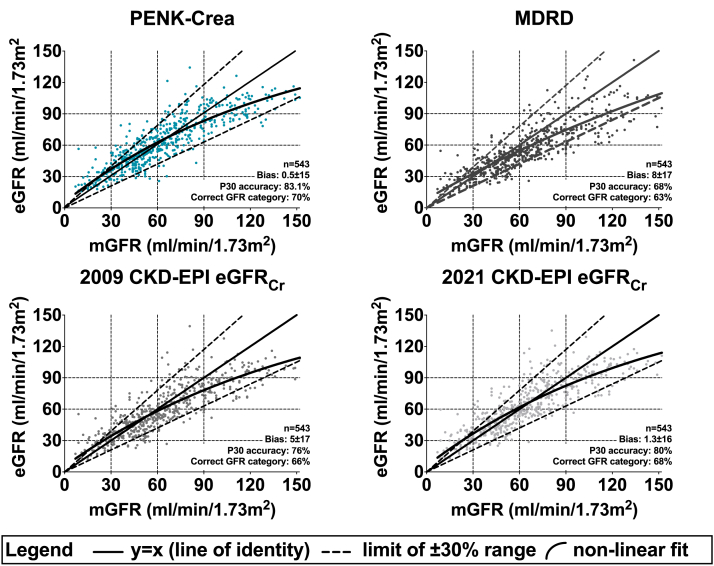
Scatter plots of the estimated GFR of all equations versus the measured GFR. Scatter plot of the association between the glomerular filtration rate measured by iohexol or iothalamate clearance (mGFR) and the eGFR calculated using the PENK-Crea equation, the conventional MDRD and 2009 CKD-EPI equations and the new 2021 CKD-EPI equation (eGFR) in the validation cohort. *Bias* is defined as the mean difference with standard deviation (in ml/min/1.73 m^2^), P30 accuracy as the percentage of estimated GFRs that is within a ±30% range of the mGFR, and correct GFR category as the percentage of patients that are correctly classified in GFR categories (all in ml/min/1.73 m^2^): “G4–5, ≤29 ml/min/1.73 m^2^,” “G3a–b, 30–59 ml/min/1.73 m^2^,” G2, “60–89 ml/min/1.73 m^2^,” and “G1, ≥90 ml/min/1.73 m^2^.” The nonlinear fit is calculated using a 2-phase association. GFR, glomerular filtration rate.

**Figure 4:**
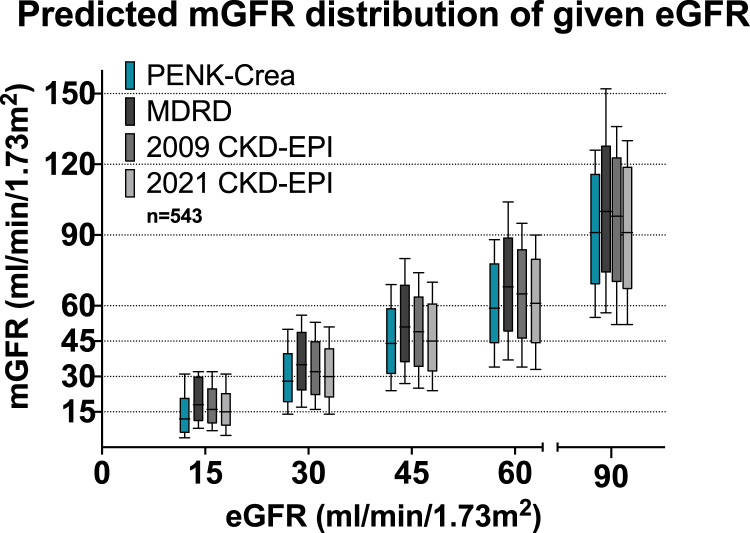
Distribution of the predicted mGFR at given eGFR cut-off points. To enable clinical decision making, this figure depicts the range of predicted mGFR of a patient with a given eGFR using the CKD staging cut-off points. The mGFR was predicted using a model created using quintile regression of each of the 4 equations PENK-Crea, MDRD, 2009 CKD-EPI and 2021 CKD-EPI. The Box Plots represent quantiles (minimum, first quartile, median, third quartile, and maximum) of each equation. The performance of an equation is better when the median of the predicted mGFR is closest to the given eGFR cut-off points, and when the distribution of the predicted mGFR is more narrow at that eGFR cut-off point. As an example, with an eGFR of 60 ml/min/1.73 m^2^ calculated with the PENK-Crea equation, 95% of the mGFR’s will be in the range of 34–88. While an eGFR of 60 calculated with the MDRD equation, 95% of the mGFR’s will be between the range of 37–104. mGFR or eGFR: measured or estimated glomerular filtration rate. CKD-EPI, Chronic Kidney Disease Epidemiology Collaboration; MDRD, Modification of Diet in Renal Disease.

The PENK-Crea equation had a mean ± SD bias of 0.5 ± 15 ml/min per 1.73 m^2^ with the mGFR significantly less compared with those of the MDRD (8±17 ml/min per 1.73 m^2^, *P* < 0.001) and 2009 CKD-EPI (5 ± 17 ml/min per 1.73 m^2^, *P* < 0.001), not reaching statistical significance compared with 2021 CKD-EPI (1.3 ± 16 ml/min per 1.73 m^2^, *P* = 0.06) (Figure 2a, Figure 3, Table 3, and Supplementary Table S2). The median [IQR] bias was −1 [−9 to 9] for PENK-Crea, 7 [−1 to 17] for MDRD, 4 [−4 to 13] for 2009 CKD-EPI, and 1 [−7 to 10] for 2021 CKD-EPI. The concordance correlation coefficient with 95% CI was 0.85 (0.82–0.87) for PENK-Crea, 0.76 (0.73–0.80) for MDRD, 0.81 (0.78–0.84) for 2009 CKD-EPI, and 0.83 (0.81–0.85) for 2021 CKD-EPI. In the Bland-Altman analysis, the limits of agreement were −29 and 30 ml/min per 1.73 m^2^ for PENK-Crea, −25 and 41 for MDRD, −26 and 35 for 2009 CKD-EPI, and −29 and 32 ml/min per 1.73 m^2^ for 2021 CKD-EPI. Linear regression in the Bland-Altman plot revealed a slope of 0.20 ± 0.02 for PENK-Crea, 0.21 ± 0.03 for MDRD, 0.23 ± 0.02 for 2009 CKD-EPI, and 0.20 ± 0.02 for 2021 CKD-EPI (Supplementary Figure S4). The P30 accuracy, calculated as the percentage of PENK-Crea eGFRs between a range of ±30% of the mGFR, was 83%, CI (80–86), significantly higher compared with MDRD (68% (64–72), *P* < 0.001) and 2009 CKD-EPI (76% (72–79), *P* < 0.001) but not significantly higher compared with 2021 CKD-EPI (80% (77–83), *P* = 0.13) (Figure 2b, Figure 3, Table 3, and Supplementary Table S2). The P20 for PENK-Crea was 62% (58–66), for MDRD 52% (48–56), for 2009 CKD-EPI 58% (54–62) and for 2021 CKD-EPI 63% (59–67). The P10 for PENK-Crea was 35% (31–39), for MDRD 27% (23–31), for 2009 CKD-EPI 31% (23–31), and for 2021 CKD-EPI 35% (31–39). For clinical decision-making, the prediction intervals describe the range of mGFR that a patient could have at an eGFR. The 95% prediction interval gives the median of the difference between the 2.5th and the 97.5th percentiles of the predicted mGFR, whereas the 50% prediction interval gives the median of the difference between the 25th and the 75th percentiles. Therefore, a smaller prediction interval is better. The 95% prediction interval for PENK-Crea was 44 ml/min per 1.73 m^2^, for MDRD it was 53, for 2009 CKD-EPI it was 49, and for 2021 CKD-EPI it was 47. The 50% prediction interval for PENK-Crea was 14 ml/min per 1.73 m^2^, for MDRD it was 16, for 2009 CKD-EPI it was 14, and for 2021 CKD-EPI it was 14 (Figure 4).

**Table 3:** Equation performance overview in all patients and subgroups

	All patients	Stable patients *n* = 456	Critically ill patients *n* = 87	CKD category G1, GFR > 90 *n* = 101	G2, GFR 89–60 *n* = 172	G3a–b, GFR 59–30 *n* = 213	G4–5, GFR<30 *n* = 57
Accuracy P30							
PENK-Crea	83 (80–86)	85 (82–88)	72 (63–81)	79 (71–87)	88 (84–93)	86 (82–91)	61 (49–74)
MDRD	68 (64–72)	68 (64–72)	69 (59–78)	47 (38–57)	70 (63–77)	75 (69–80)	65 (53–77)
2009 CKD-EPI	76 (72–79)	77 (73–80)	69 (59–78)	65 (56–75)	82 (76–88)	77 (71–83)	68 (56–80)
2021 CKD-EPI	80 (77–83)	80 (76–83)	82 (73–89)	87 (81–94)	87 (82–92)	79 (74–85)	49 (36–62)
Bias and precision, mean ± SD							
PENK-Crea	0.5 ± 15	−1 ± 13	8 ± 20	16 ± 14	0 ± 15	−3 ± 11	−8 ± 10
MDRD	8 ± 17	7 ± 15	9 ± 24	24 ± 19	8 ± 17	3 ± 10	6 ± 9
2009 CKD-EPI	5 ± 17	4 ± 14	11 ± 20	22 ± 15	4 ± 15	1 ± 11	−6 ± 9
2021 CKD-EPI	1.3 ± 16	0.5 ± 15	6 ± 19	17 ± 15	1 ± 15	−2 ± 12	−8 ± 10

CKD, chronic kidney disease; CKD-EPI, chronic kidney disease-epidemiology collaboration; MDRD, modification of diet in renal disease.

Bias and precision were calculated using mGFR minus eGFR, thus a positive bias represents an underestimation, and depicted in mean ± SD bias in ml/min/1.73 m^2^. The P30 accuracy represents the percentage of eGFR that is within a range of ±30% of the mGFR, and is depicted in percentages with 95% confidence interval.

To further look into the difference in P30 accuracy in different GFR categories, GFR was staged by mGFR according to the KDIGO CKD categories,^8^ which were combined to prevent small groups of patients. The PENK-Crea equation was more accurate compared with MDRD and 2009 CKD-EPI in GFR categories G1, G2, and G3a and b, whereas for 2021 CKD-EPI that was the case in G3a and b. “G1, ≥90 ml/min per 1.73 m^2^” (MDRD: *P* < 0.001, 2009 CKD-EPI: *P* = 0.02, and 2021 CKD-EPI: *P* = 0.10), “G2, 60 to 89 ml/min per 1.73 m^2^” (MDRD: *P* < 0.001, 2009 CKD-EPI: *P* = 0.04, and 2021 CKD-EPI: *P* = 0.86), and “G3a to b, 30 to 59 ml/min per 1.73 m^2^” (MDRD: *P* < 0.001, 2009 CKD-EPI: *P* = 0.002, and 2021 CKD-EPI: *P* = 0.03). There was no statistically significant difference between PENK-Crea and the creatinine-based equations in category “G4 to 5, ≤29 ml/min per 1.73 m^2^” (MDRD: *P* = 0.77, 2009 CKD-EPI: *P* = 0.39, and 2021 CKD-EPI: *P* = 0.09) (Figure 2c, Table 3, and Supplementary Table S2). For bias and precision according to CKD category, see Table 3 and Supplementary Table S2.

#### Validation in Stable Patients

In 456 patients with a stable kidney function, the PENK-Crea equation had a mean ± SD bias of −1 ± 13 ml/min per 1.73 m^2^ compared with the mGFR, significantly less compared with those of the MDRD (7 ± 15 ml/min per 1.73 m^2^, *P* < 0.001) and 2009 CKD-EPI (4 ± 14 ml/min per 1.73 m^2^, *P* < 0.001), and while statistically significant, not clinically relevant different compared with 2021 CKD-EPI (1 ± 15 ml/min per 1.73 m^2^, *P* = 0.003) (Supplementary Figure S5). The median [IQR] bias was −2 [−9 to 6] for PENK-Crea, 6 [−1 to 15] for MDRD, 3 [−5 to 11] for 2009 CKD-EPI, and 0 [−8 to 8] for 2021 CKD-EPI. In the Bland-Altman analysis, the limits of agreement were −27 and 25 ml/min per 1.73 m^2^ for PENK-Crea, −22 and 37 for MDRD, −25 and 32 for 2009 CKD-EPI, and −28 and 29 for 2021 CKD-EPI. A linear regression revealed a slope of 0.15 ± 0.03 for PENK-Crea, 0.32 ± 0.03 for MDRD, 0.20 ± 0.03 for 2009 CKD-EPI, and 0.18 ± 0.03 (Supplementary Figure S6). The accuracy, calculated as the percentage of the PENK-Crea eGFRs between a range of ±30% of the mGFR was 85% (82–88), significantly higher compared with MDRD (68% (64–72), *P* < 0.001), 2009 CKD-EPI (77 (73–81)%, *P* < 0.001) and higher than the 2021 CKD-EPI (80% (76–83), *P* = 0.01), whereas the 95% CI is partly overlapping (Supplementary Figure S5). For bias and precision according to CKD category, see Table 3 and Supplementary Table S2.

#### Validation in Critically Ill Patients

In 87 critically ill patients, the PENK-Crea had a mean ± SD bias of 8 ± 20 ml/min per 1.73 m^2^ with the mGFR, significantly less than the 2009 CKD-EPI (11 ± 20, *P* = 0.008), similar compared with the MDRD (9 ± 24, *P* = 0.63) (Supplementary Figure S5) and higher compared with the 2021 CKD-EPI (6 ± 20 ml/min per 1.73 m^2^, *P* = 0.01). The median [IQR] bias was 12 [−3 to 21] for PENK-Crea, 10 [−2 to 24] for MDRD, 11 [1–24] for 2009 CKD-EPI, and 6 [−4 to 18] for 2021 CKD-EPI. The accuracy, calculated as the percentage of eGFRs between a range of ±30% of the mGFR, was not statistically significant between the equations: 72% (63%–81%) for PENK-Crea, compared with 69% (59%–79%) for MDRD, *P* = 0.63, 69% (59%–79%) for 2009 CKD-EPI, *P* = 0.61, and 82% (73%–90%) for 2021 CKD-EPI, *P* = 0.10 (Supplementary Figure S5). In the Bland-Altman analysis, the limits of agreement were −31 and 48 ml/min per 1.73 m^2^ for PENK-Crea, −39 and 56 for MDRD, −28 and 50 for 2009 CKD-EPI, and −32 and 44 for 2021 CKD-EPI. A linear regression revealed a slope of 0.35 ± 0.09 for PENK-Crea, 0.03 ± 0.11 for MDRD, 0.39 ± 0.09 for 2009 CKD-EPI, and 0.38 ± 0.09 for 2021 CKD-EPI (Supplementary Figure S6). For bias and precision according to CKD category, see Table 3 and Supplementary Table S2.

## Discussion

In this study, a novel equation to assess eGFR using proenkephalin was developed and validated in a broad cohort including healthy organ donors, stable outclinic patients, patients with CKD, and critically ill patients. The new PENK-Crea equation to calculate the eGFR performed better than widely used creatinine-based equations, both in bias and accuracy. Overall performance was similar to or better than the newly developed 2021 CKD-EPI equation. Finally, the addition of sex and race did not further improve the performance of the equation.

Multiple large observational studies have revealed that increases in PENK concentration predict and are associated with the occurrence of acute kidney injury.^13^^,^^35^ Given the association of renal dysfunction with unfavorable clinical outcomes,^36^ this association may explain why values of PENK, when measured at hospital or intensive care unit admission, also exert predictive value for mortality.^14^^,^^37^^,^^38^ However, the value of PENK to reflect the actual GFR has only been described sparsely and mostly in small cohorts.^17^^,^^21^^,^^39^ In the present study, for the first time, PENK was correlated with gold standard GFR measurements, in a large cohort of patients, with kidney functions ranging from low to high GFR values and from stable to critically ill patients. Despite previous indications of better performance of PENK to estimate renal function, no equation to calculate eGFR based on PENK concentrations was available up to now. This equation may facilitate biomarker implementation^40^ and translate PENK concentrations that the clinician may not be familiar with into a more accurate GFR estimation for physicians to work with. This also allows easier comparison to other functional biomarkers that reflect GFR, such as creatinine and cystatin C.

The analyses for covariate selection for the equation revealed that PENK concentration was most strongly correlated to mGFR and resulted for the larger part in the narrowing of the root mean square error of the equation, which underlines the more accurate reflection of PENK of the GFR. The addition of creatinine and age resulted in further relevant improvements. The MDRD and 2009 CKD-EPI equations also include sex and race. The use of the latter is controversial,^3^^,^^41^ and therefore the new 2021 CKD-EPI equation was developed. The 2021 CKD-EPI equation without race performed better than the 2009 CKD-EPI in our cohort, whereas PENK-Crea overall performance was better or similar compared with 2021 CKD-EPI, noteworthy in patients with a GFR in the 30–60 ml/min per 1.73 m^2^. Especially in this range, dose adjusting of renally cleared drugs becomes relevant, especially in critically ill patients or hospitalized patients with CKD prone to develop acute-on-chronic kidney injury.

In critically ill patients, it is notoriously unreliable to accurately assess kidney function based on creatinine, as active tubular secretion of creatinine may conceal acute decreases in GFR. Furthermore, critically ill patients lose muscle mass within days, leading to reduced creatinine production and overestimating the eGFR. Proenkephalin avoids these issues^16^; therefore, they may account for the observed differences between proenkephalin and creatinine-based equations. The mGFR of critically ill patients was predominantly higher compared with the mGFR of the stable patients probably because only a proportion of patients had acute kidney injury and other patients may have had an augmented renal clearance during a hyperdynamic circulation. Furthermore, a significant proportion of the stable patients were diagnosed with having CKD which was not the case for the critically ill patients. In the critically ill patients, the mGFR was measured shortly after sepsis diagnosis or shortly after cardiac surgery. Therefore, the underestimation of both the novel PENK-Crea and creatinine-based calculations of the eGFR in the critically ill patients may illustrate the acknowledged lag time of creatinine as a biomarker for GFR. Therefore, further development and validation of the equation using PENK without creatinine may be of interest for critically ill patients.

A limitation of our study is that the PENK-Crea equation has yet to be validated in larger cohorts of critically ill patients, as the number of critically ill patients in our validation cohort was relatively small. Nevertheless, a strength of this study is that the cohort used for equation development and validation included a broad range of patients, including healthy potential kidney donors, patients with established CKD, and critically ill patients. Consequently, the equation can be applied to a wide range of patients. Second, gold standard methods to determine the true mGFR were used in all patients of the cohort. Furthermore, the data-driven approach applied to develop the equation ensures an unbiased selection of covariates. However, even though this cohort consisted of multiple patient types and was block randomized in a blinded fashion to create a representative internal validation cohort, the performance of the PENK-Crea equation is still to be validated in external cohorts.

Although both exogenous compounds iohexol and iothalamate are considered the most accurate methods to measure GFR,^10^^,^^20^ a cohort with only 1 gold standard method to assess the GFR would have been more optimal. Unfortunately, it is difficult to identify large cohorts with mGFR, especially outside clinical investigation or with a broad range of patients; subsequently, most equations, including CKD-EPI equations, include data on mGFR by multiple methods.^3^^,^^27^^,^^28^ A direct comparison between the 2 gold-standard methods is hampered by the fact that iohexol was used in critically ill patients and iothalamate clearance in stable patients in our study. Although our cohort provided subgroups large enough for covariate eligibility testing, a limitation of this study is that the subgroups differ in size. Furthermore, a comparison to equations using cystatin C, which can be accurate in specific patient groups,^42^ is of interest. However, several factors that influence cystatin C production, such as corticosteroid use and inflammation,^43^^,^^44^ are acknowledged, and cystatin C and its equations are not frequently used in clinical practice. Especially in critically ill patients, corticosteroids and inflammations that may influence the accuracy of cystatin C are present in many patients. Therefore, we compared PENK-Crea to the widely used creatinine-based equations. Nevertheless, the absence of comparison with a cystatin C-based equation is a limitation of this study, and further testing of the PENK-Crea equation in cohorts also including cystatin C measurements is warranted. Finally, to study the possible benefit of more precise and accurate estimation of the GFR by the PENK-Crea equation in clinical practice will require pharmacokinetic studies that, for example, assess renally cleared drugs.^45^ Especially in critically ill patients, demonstration that a more precise calculation of eGFR will translate to a clinical outcome benefit will be challenging, although consensus guidelines^46^, ^47^, ^48^ make it clear that a more timely and accurate estimation of GFR is a relevant unmet medical need.

In conclusion, we developed an equation to calculate eGFR using the novel biomarker for kidney function PENK, with creatinine and age. Overall, this PENK-Crea equation performs better to calculate GFR compared with most conventional or more novel creatinine-based equations. Furthermore, the frequently used covariates sex and race, of which the latter is under scrutiny, did not further improve the performance of the equation and are therefore not included in the PENK-Crea equation. These results warrant validation in external patient cohorts that will facilitate future studies into possible clinical benefits of more accurate GFR measurement.

## Disclosure

RB has received a travel reimbursement from SphingoTec. PP has received travel and consultancy reimbursement from SphingoTec. JCL has received grants from Allena, Arkray, Siemens, and Retrophin; others from Federation Bio, Novobiome, and Orfan-Bridgebio; and grants and others from Alnylam Pharmaceuticals, Dicerna Pharmaceuticals, OxThera, and Synlogic. ASJ presently or has in the past consulted for most of the major diagnostic companies and SphingoTec. CCW, JS, and BA are employed by SphingoTec GmbH. The proenkephalin measurements were conducted by SphingoTec, GmbH. The remaining authors declare no competing interests.

## Supplementary Material

Supplementary File (PDF)

**Figure S1.** Scatter plots of proenkephalin and creatinine with the measured GFR.

**Figure S2.** Contour plots for PENK-CR.

**Figure S3.** Contour plots for 2021 CKD-EPI.

**Figure S4.** Bland-Altman plots.

**Figure S5.** Performance PENK-Crea, MDRD, and CKD-EPI formulas in subgroups.

**Figure S6.** Bland-Altman plots of PENK-Crea and creatinine-based equations in subgroups.

**Table S1.** Performance of the PENK-only, European Kidney Function Consortium, and revised Lund-Malmö equations.

**Table S2.** Equation performance overview in subgroups.

**STROBE Statement**.
