# Supplementary material for: Assessing GFR With Proenkephalin
Source: Kidney Int Rep. 2023 Aug 19;8(11):2345–55. doi: 10.1016/j.ekir.2023.08.006 (PMC10658254; doi:10.1016/j.ekir.2023.08.006)
Supplement: Supplementary File (PDF) [file mmc1.pdf]

Supplemental results from manuscript:

Assessing Glomerular Filtration Rate with Proenkephalin

Authors

Remi Beunders MSc[1,2], Leslie J Donato PhD[3], Roger van Groenendael MSc[1,2,5], Birte Arlt PhD[6], Cristiane Carvalho-Wodarz PhD[6], Janin Schulte PhD[6], Prof Anton CC Coolen[7,8], Prof John C Lieske, PhD[3,4], Jeffrey W Meeusen PhD[3], Prof Allan S Jaffe[3,9] and Prof Peter Pickkers[1,2]

Corresponding author and address for reprints.

Peter Pickkers, MD, PhD. Department of Intensive Care Medicine, Radboud University Medical Center, PO Box 9101, NL 6500 HB, Nijmegen, The Netherlands. Email: peter.pickkers@radboudumc.nl. Phone: +31 2436 17273.

Content

Results 3

Supplement Figure S1. Scatter plots of proenkephalin and creatinine with the measured GFR........ 3

Supplement Figure S2. Contour plots for PENK-Crea.. 4

Supplement Figure S3. Contour plots for 2021 CKD-EPI 6

Supplement Figure S4. Bland-Altman plots... 8

Supplement Figure S5. Performance PENK-Crea, MDRD and CKD-EPI equations in subgroups......... 9

Supplement Figure S6. Bland-Altman plots of PENK-Crea and creatinine-based equations in subgroups 10

Supplement Table S1. Performance of the PENK-only equation 12

Supplement Table S2. Equation performance overview in subgroups 13

Results

Supplement Figure S1. Scatter plots of proenkephalin and creatinine with the measured GFR

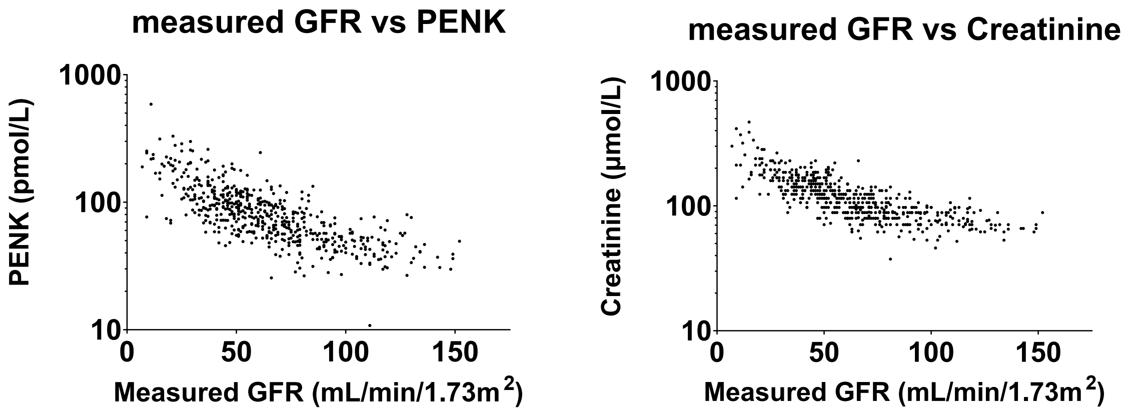

Scatter plots of the measured GFR with proenkephalin (*Pearson's r* = -0.76, p<0.001) on the left and creatinine on the right (*Pearson's r* =-0.75, p<0.001). Biomarker data is presented on a logarithmic y-axis, given the logarithmic representation of the data used in the equation. Conversion factors for serum creatinine between mg/dL to µmol/L: 88.42 GFR: glomerular filtration rate

Supplement Figure S2. Contour plots for PENK-Crea

A

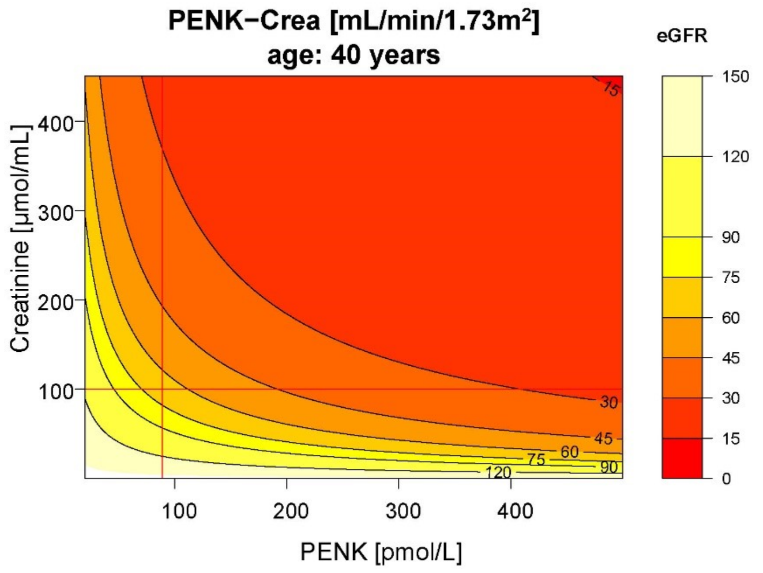

B

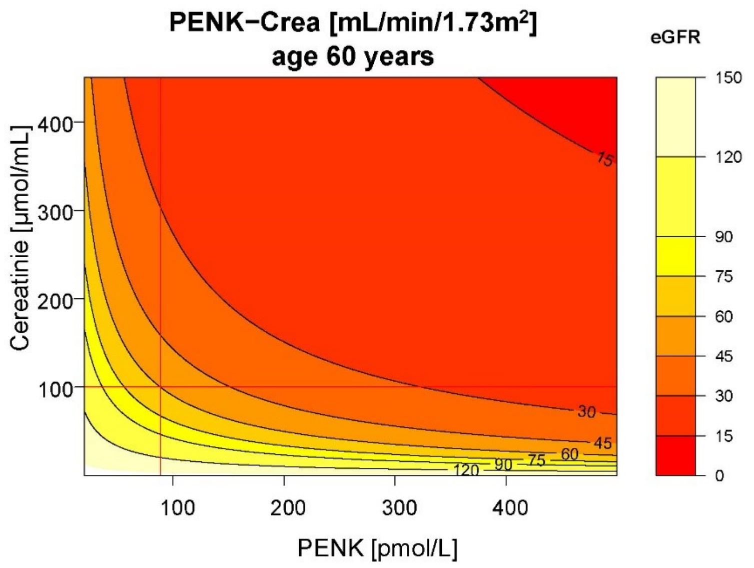

C

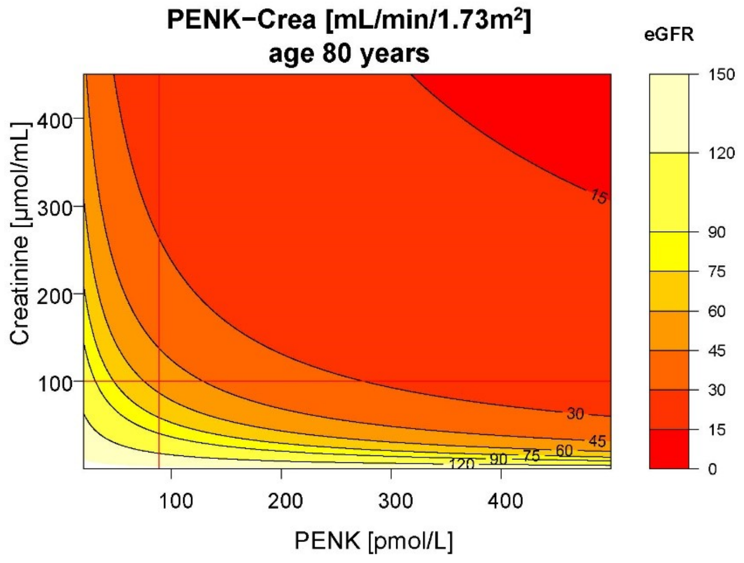

Contour plots for PENK-Crea (based on PENK, creatinine, and age) to illustrate the impact of each parameter in the PENK-Crea formula on the calculated eGFR. We have selected PENK and creatinine for the two axes and fixed age at A) 40 years, B) 60 years, and C) 80 years (left to right). The resulting GFR estimate is displayed at the respective x/y-coordinate by color code (see the legend on the right) by category <15, 15–30, 30–45, 45–60, 60–75, 75–90, 90–120 and >120. Contour lines mark the respective GFR cut points (15, 30, 45, 60, 75, 90, 120). Example: For a 40-year-old patient with a PENK concentration of 89 pmol/L and a creatinine of 100 µmol/L, the PENK-Crea formula estimates the GFR at 60 mL/min/1.73m^2. For a patient age 80 years with identical PENK and creatinine concentration, the GFR would be lower (45–60 mL/min/1.73m^2), and for a patient age 40, it would be higher (75–90 mL/min/1.73m^2).

Supplement Figure S3. Contour plots for 2021 CKD-EPI

A

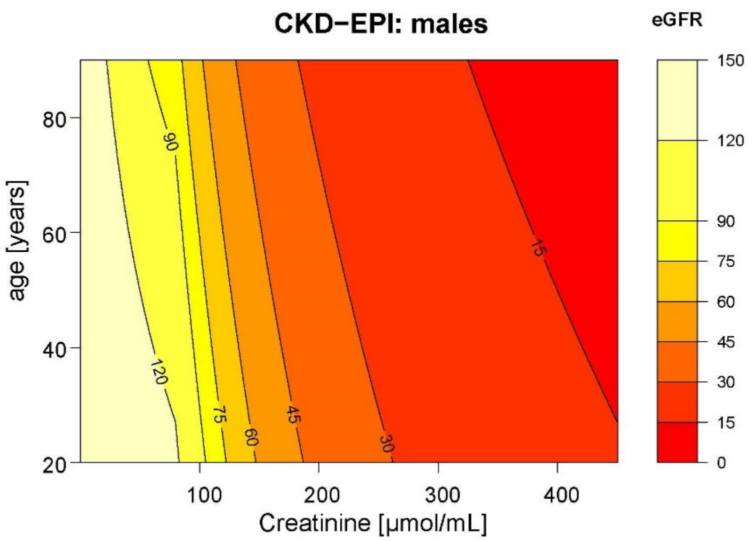

B

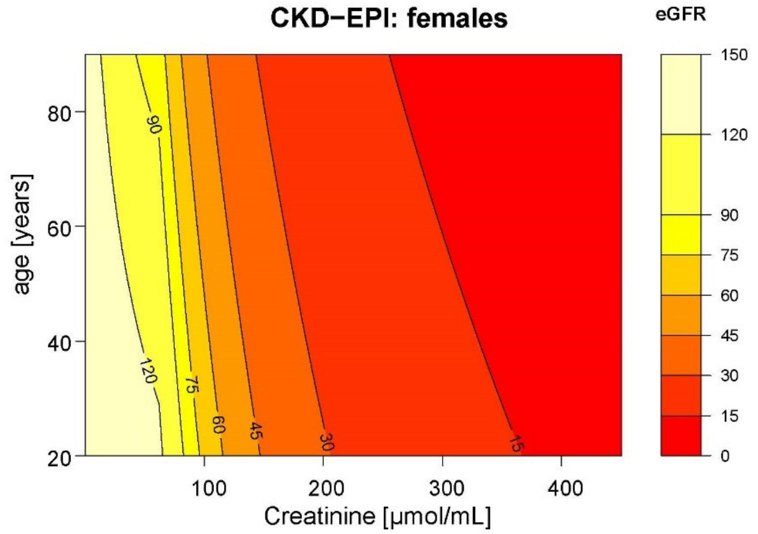

Contour plots for $eGFR_{CKD-EPI}$ (2021, based on creatinine, age and sex), to illustrate the impact of each parameter in the PENK-Crea formula on the calculated eGFR. We have selected creatinine and age for the two axes, and provide separate plots for males (A) and females (B). The resulting GFR estimate is displayed at the respective x/y-coordinate by color code (see legend on the right) by category <15, 15–30, 30–45, 45–60, 60–75, 75–90, 90–120 and >120. Contour lines mark the respective GFR cut points (15, 30, 45, 60, 75, 90, 120). Example: For a 60-year old male patient with a creatinine concentration of 177 µmol/L the CKD-EPI formula estimates the GFR between

30–45 mL/min/1.73m^2. For a female patient age 60 years with identical creatinine concentration, the GFR would be lower (15–30 mL/min/1.73m^2).

Supplement Figure S4. Bland-Altman plots

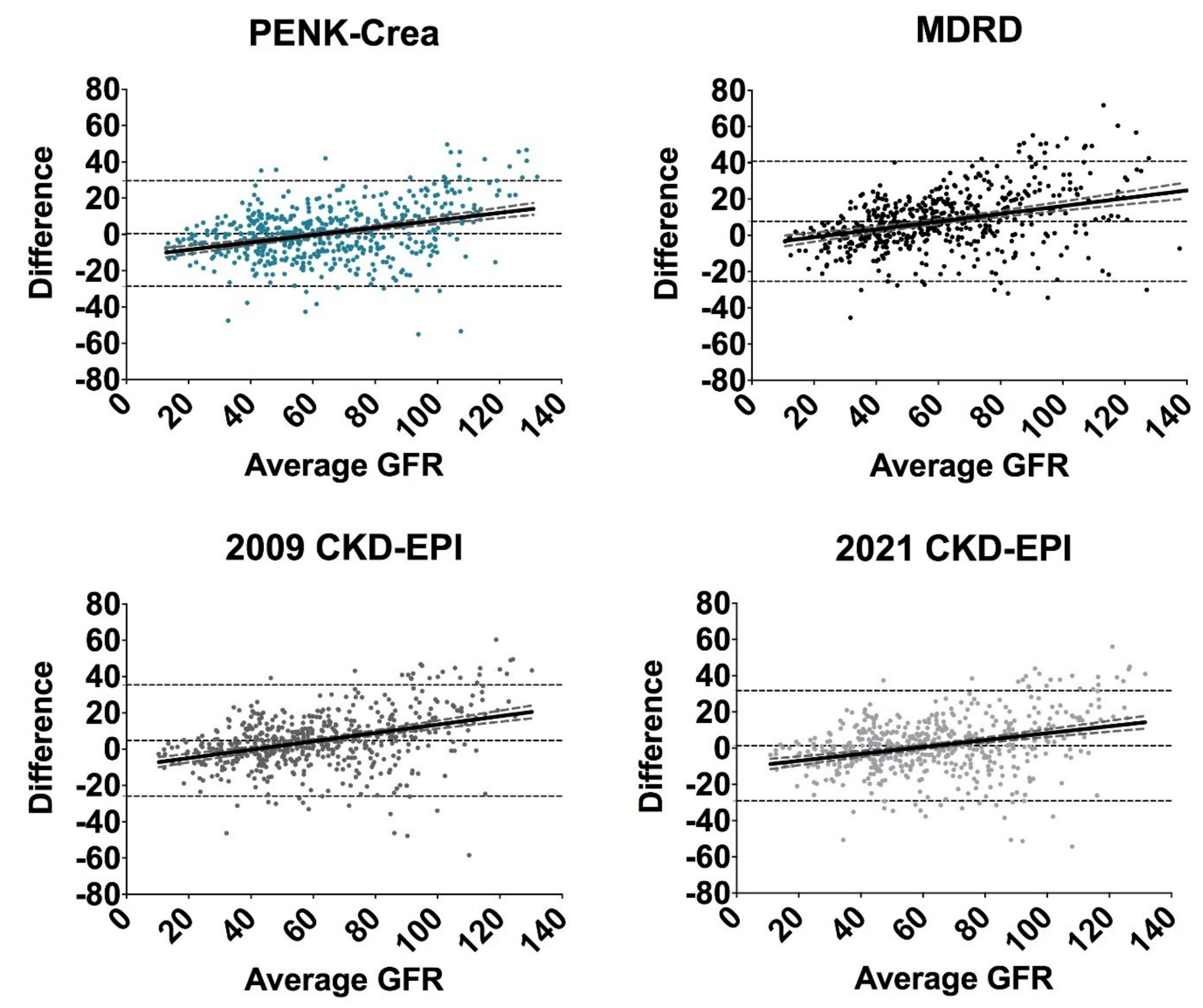

Bland-Altman plots of all equations and the measured GFR. On the y-axis the difference between the predicted and measured GFR in mL/min/1.73m^2. On the x-axis the average of the predicted and true GFR. Furthermore a least-squares linear regression line with 95% confidence interval is shown. GFR: glomerular filtration rate, CKD-EPI: Chronic Kidney Disease Epidemiology Collaboration.

Supplement Figure S5. Performance PENK-Crea, MDRD and CKD-EPI equations in subgroups

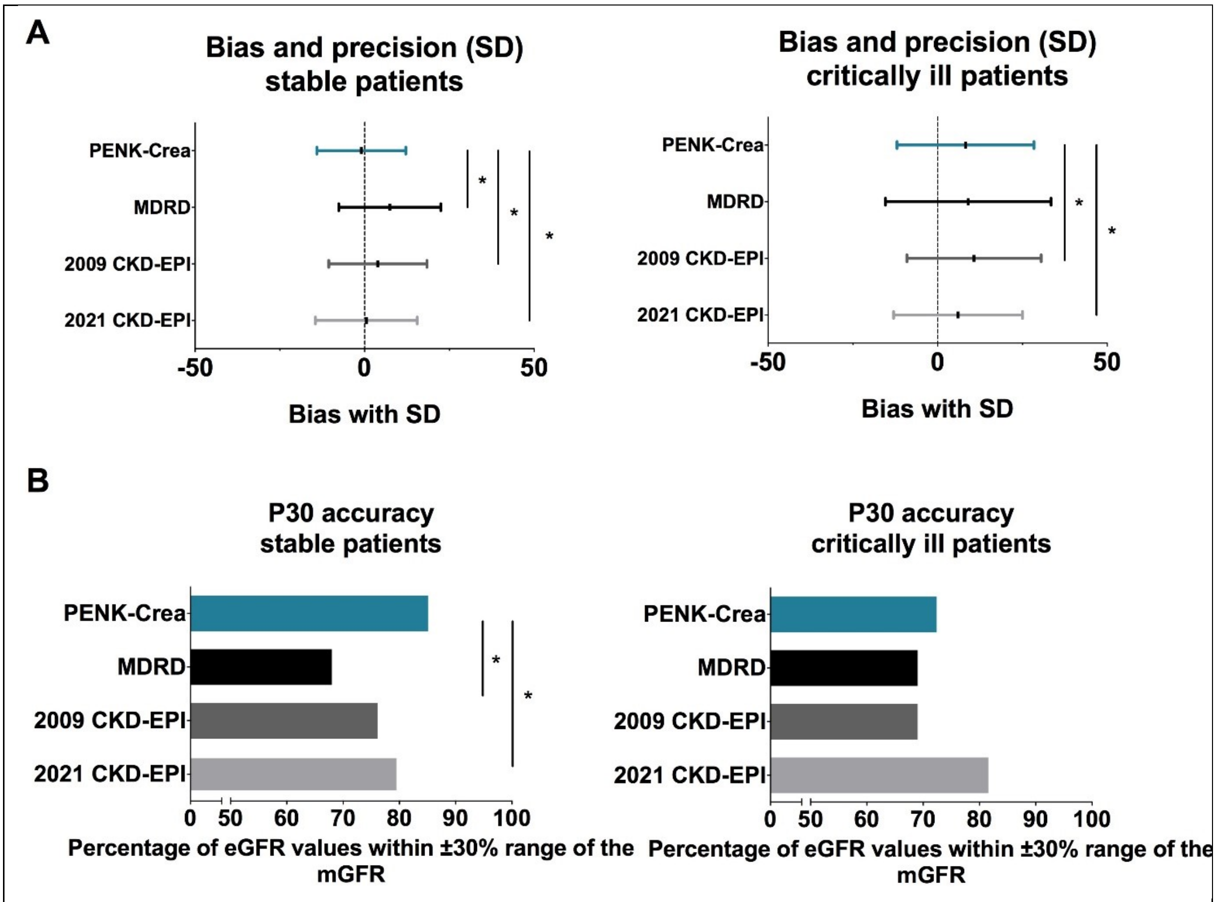

An overview of the performance of the PENK-Crea and the MDRD and CKD-EPI equations in the validation cohort.

A. Mean bias in mL/min/1.73m^2 with standard deviation (SD) from the measured GFR of the equations, with on the left: tested in patients with a steady state kidney function and on the right: tested in patients with a non-steady state kidney function.

B. Accuracy (proportion of estimated GFR that is within ±30% of the measured GFR) of the equations, with on the left: tested in patients with a steady state kidney function and on the right: tested in patients with a non-steady state kidney function. GFR: glomerular filtration rate. *: $p<0.05$.

Supplement Figure S6. Bland-Altman plots of PENK-Crea and creatinine-based equations in subgroups

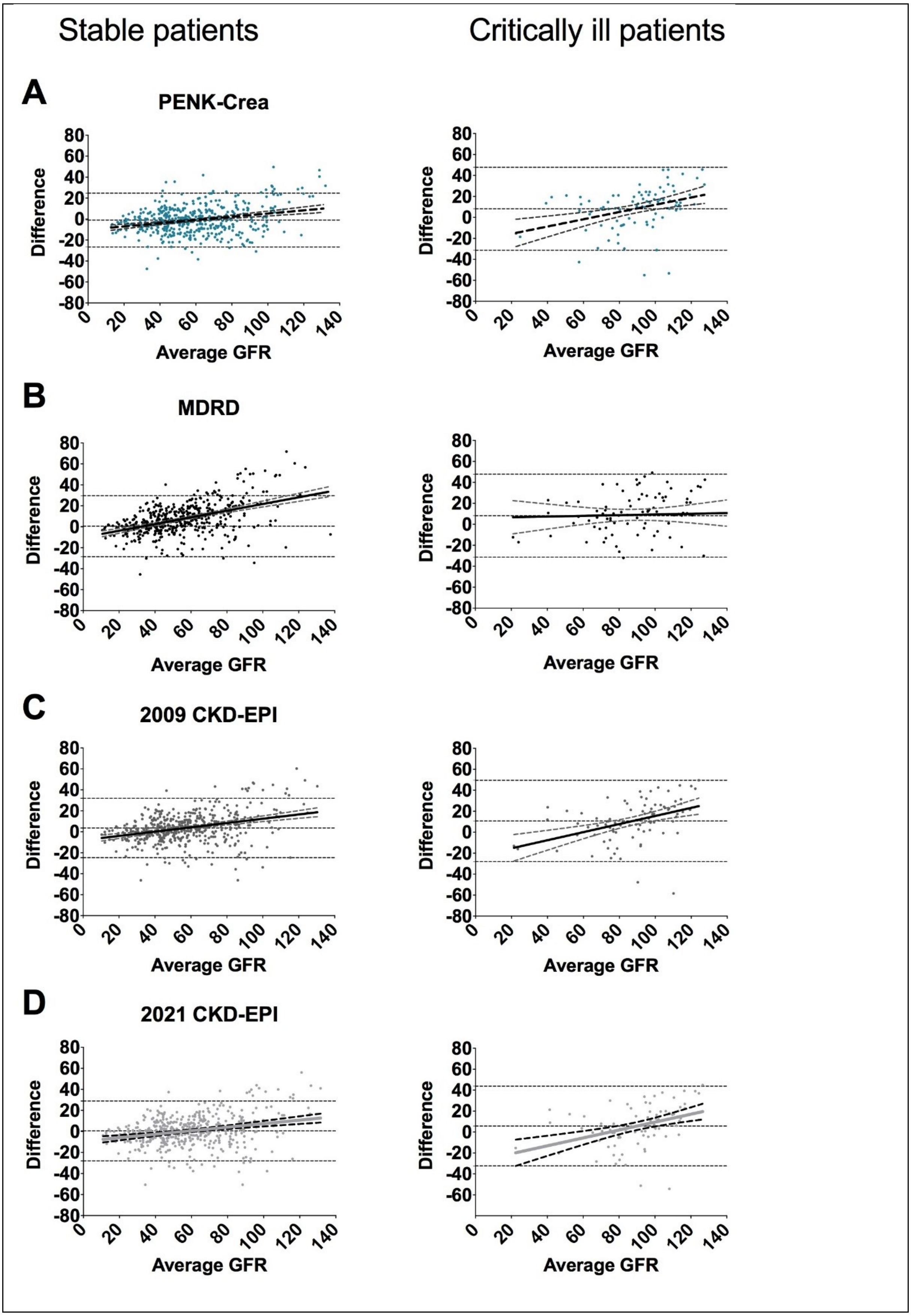

Bland-Altman plots with on the y-axis the difference in mL/min/1.73m^2 between the predicted and measured GFR (mGFR). On the x-axis the average of the predicted and mGFR. Depicted in patients ith steady state kidney function (stable, n=456) on the left and non-steady state kidney function on the right (critically ill, n=87). **A** shows PENK-based PENK-Crea equation, **B** shows the creatinine-based MDRD equation, **C** shows the conventional creatinine-based 2009 CKD-EPI equation and **D** shows the novel creatinine-based 2021 CKD-EPI equation. Furthermore a least-squares linear regression line with 95% confidence interval are depicted. GFR: glomerular filtration rate.

Supplement Table S1. Performance of the PENK-only, European Kidney Function Consortium and revised Lund-Malmö equations

	All patients n=543	Stable patients n=456	Critically ill patients n=87
**Mean±SD bias in (ml/min/1.73m^2)**			
PENK-only	-3±20	-0±18	-15±25
European Kidney Function Consortium	-7±16	-5±15	-16±20
revised Lund-Malmö	-10±17	-9±16	-18±21
**P30 accuracy (%)**			
PENK-only	70 (66–74)	70 (66–75)	68 (58–78)
European Kidney Function Consortium	80 (77–83)	81 (78–85)	75 (66–84)
revised Lund-Malmö	71 (67–74)	72 (68–77)	61 (51–71)

Performance was tested in the validation cohort. Bias and precision were calculated using mGFR minus eGFR, thus a positive bias represents an underestimation, and depicted in mean±standard deviation bias in ml/min/1.73m^2. The P30 accuracy represents the percentage of eGFR that is within a range of ±30% of the mGFR, and is depicted in percentages with 95% confidence interval.

Supplement Table S2. Equation performance overview in subgroups

	**CKD category G1, GFR>90 n=101**	**G2, GFR 89–60 n=172**	**G3a–b, GFR 59–30 n=213**	**G4–5, GFR<30 n=57**
**Accuracy P30**	Stable n=53<br>Critically ill n=48	Stable n=140<br>Critically ill n=32	Stable n=208<br>Critically ill n=5	Stable n=55<br>Critically ill n=2
**PENK-Crea**				
Stable patients	*81 (71-92)*	*91 (86-96)*	*88 (84-92)*	*64 (51-76)*
Critically ill patients	*77 (65-89)*	*78 (64-92)*	*	*
**MDRD**				
Stable patients	*57 (43-70)*	*67 (59-75)*	*75 (69-81)*	*67 (55-80)*
Critically ill patients	*37 (24-51)*	*84 (72-97)*	*	*
**2009 CKD-EPI**				
Stable patients	*64 (51-77)*	*81 (75-88)*	*78 (73-84)*	*71 (89-83)*
Critically ill patients	*67 (53-80)*	*84 (72-97)*	*	*
**2021 CKD-EPI**				
Stable patients	*81 (71-92)*	*90 (85-95)*	*80 (75-86)*	*51 (38-64)*
Critically ill patients	*94 (87-101)*	*75 (60-90)*	*	*
**Bias and precision, mean±SD**	Stable n=53<br>Critically ill n=48	Stable n=140<br>Critically ill n=32	Stable n=208<br>Critically ill n=5	Stable n=55<br>Critically ill n=2
**PENK-Crea**				
Stable patients	*13±15*	*0±13*	*-4±10*	*-8±10*
Critically ill patients	*19±13*	*-3±20*	*	*
**MDRD**				
Stable patients	*29±18*	*11±13*	*3±10*	*-6±9*
Critically ill patients	*19±18*	*-3±29*	*	*
**2009 CKD-EPI**				
Stable patients	*22±16*	*5±14*	*1±11*	*-6±9*
Critically ill patients	*22±13*	*-2±19*	*	*
**2021 CKD-EPI**				
Stable patients	*18±17*	*1±14*	*-2±11*	*-8±10*
Critically ill patients	*17±13*	*-7±18*	*	*

*Bias and precision were calculated using mGFR-eGFR, thus a positive bias represents an underestimation, and depicted in mean±standard deviation bias in ml/min/1.73m^2. The P30 accuracy represents the percentage of eGFR that is within a range of ±30% of the mGFR, and is depicted in percentages with 95% confidence interval. * because of small subgroups of n=5 and n=2, the statistics were not meaningful and are not shown. CKD: chronic kidney disease. MDRD: Modification of Diet in Renal Disease, CKD-EPI: Chronic Kidney Disease - Epidemiology Collaboration.*

STROBE Statement—Checklist of items that should be included in reports of ***cohort studies***

	Item No	Recommendation	Page No
**Title and abstract**	1	(*a*) Indicate the study's design with a commonly used term in the title or the abstract<br>(*b*) Provide in the abstract an informative and balanced summary of what was done and what was found	0, 2
**Introduction**			
Background/rationale	2	Explain the scientific background and rationale for the investigation being reported	3
Objectives	3	State specific objectives, including any prespecified hypotheses	3
**Methods**			
Study design	4	Present key elements of study design early in the paper	4
Setting	5	Describe the setting, locations, and relevant dates, including periods of recruitment, exposure, follow-up, and data collection	4
Participants	6	(*a*) Give the eligibility criteria, and the sources and methods of selection of participants. Describe methods of follow-up<br>(*b*) For matched studies, give matching criteria and number of exposed and unexposed	(a) 4<br>(b) n.a.
Variables	7	Clearly define all outcomes, exposures, predictors, potential confounders, and effect modifiers. Give diagnostic criteria, if applicable	5, 6
Data sources/ measurement	8*	For each variable of interest, give sources of data and details of methods of assessment (measurement). Describe comparability of assessment methods if there is more than one group	5, 6
Bias	9	Describe any efforts to address potential sources of bias	5, 6
Study size	10	Explain how the study size was arrived at	4
Quantitative variables	11	Explain how quantitative variables were handled in the analyses. If applicable, describe which groupings were chosen and why	4
Statistical methods	12	(*a*) Describe all statistical methods, including those used to control for confounding<br>(*b*) Describe any methods used to examine subgroups and interactions<br>(*c*) Explain how missing data were addressed<br>(*d*) If applicable, explain how loss to follow-up was addressed<br>(*e*) Describe any sensitivity analyses	5, 6
**Results**			
Participants	13*	(a) Report numbers of individuals at each stage of study—eg numbers potentially eligible, examined for eligibility, confirmed eligible, included in the study, completing follow-up, and analysed	(a) 7<br>(b) n.a.

		(b) Give reasons for non-participation at each stage<br>(c) Consider use of a flow diagram	(c) n.a.
Descriptive data	14*	(a) Give characteristics of study participants (eg demographic, clinical, social) and information on exposures and potential confounders<br>(b) Indicate number of participants with missing data for each variable of interest<br>(c) Summarise follow-up time (eg, average and total amount)	(a) 7, table 1<br>(b) n.a.<br>(c) n.a.
Outcome data	15*	Report numbers of outcome events or summary measures over time	n.a
Main results	16	(*a*) Give unadjusted estimates and, if applicable, confounder-adjusted estimates and their precision (eg, 95% confidence interval). Make clear which confounders were adjusted for and why they were included<br>(*b*) Report category boundaries when continuous variables were categorized<br>(*c*) If relevant, consider translating estimates of relative risk into absolute risk for a meaningful time period	(a) 7–11<br>(b, c) n.a.
Other analyses	17	Report other analyses done—eg analyses of subgroups and interactions, and sensitivity analyses	7–11, tables, figures and suppl. results
**Discussion**			
Key results	18	Summarise key results with reference to study objectives	11
Limitations	19	Discuss limitations of the study, taking into account sources of potential bias or imprecision. Discuss both direction and magnitude of any potential bias	12, 13
Interpretation	20	Give a cautious overall interpretation of results considering objectives, limitations, multiplicity of analyses, results from similar studies, and other relevant evidence	11–14
Generalisability	21	Discuss the generalisability (external validity) of the study results	12–14
**Other information**			
Funding	22	Give the source of funding and the role of the funders for the present study and, if applicable, for the original study on which the present article is based	15, 16

*Give information separately for exposed and unexposed groups.

Note: An Explanation and Elaboration article discusses each checklist item and gives methodological background and published examples of transparent reporting. The STROBE checklist is best used in conjunction with this article (freely available on the Web sites of PLoS Medicine at http://www.plosmedicine.org/, Annals of Internal Medicine at http://www.annals.org/, and Epidemiology at http://www.epidem.com/). Information on the STROBE Initiative is available at http://www.strobe-statement.org.
